# Perioperative red blood cell infusion and deep vein thrombosis in patients with femoral and pelvic fractures: a propensity score matching

**DOI:** 10.1186/s13018-021-02510-6

**Published:** 2021-06-05

**Authors:** Linqin Wu, Bo Cheng

**Affiliations:** Department of Anesthesiology, The First Affiliated Hospital of Chongqing Medical University, Chongqing, China

**Keywords:** Red blood cell infusion, Femoral fracture, Pelvic fracture, Deep vein thrombosis, Propensity score matching

## Abstract

**Background:**

The relationship between perioperative red blood cell (RBC) infusion and deep vein thrombosis (DVT) has not been determined.

**Objectives:**

To analyze the time-event relationship between perioperative RBC infusion and DVT in patients with femoral and pelvic fractures after adjusting for confounding factors and to provide reference for optimizing DVT risk factors.

**Methods:**

The clinical data of 569 patients with femoral and pelvic fractures who received surgical treatment from May 2018 to December 2019 were retrospectively analyzed. Propensity score matching (PSM) was performed on 20 covariates of DVT. With the formation or progression of DVT after RBC infusion as the end point, the time-event relationship between perioperative RBC infusion and DVT in patients was analyzed by binary logistic regression.

**Results:**

After 1:1 PSM of 569 patients included in this study, 126 patients were in the transfusion group and the non-transfusion group, respectively. Before PSM (*P* = 0.023, OR = 1.496 [95% CI, 1.058-2.115]), perioperative RBC infusion was associated with DVT formation for femoral and pelvic fractures. This conclusion was still obtained after PSM (*P* = 0.038, OR = 1.728, 95% CI = (1.031, 2.896)). The risk of DVT in patients with RBC infusion of 2-4U and > 4U is 1.833 and 2.667 times that of ≤ 2U, respectively. After excluding patients who received preoperative RBC infusion and had DVT formation or progression prior to RBC infusion, perioperative RBC infusion was still associated with the formation of DVT in femoral and pelvic fractures (*P* = 0.037, OR = 2.231 [95% CI, 1.049-4.745]).

**Conclusion:**

Perioperative RBC infusion is one of the causes of DVT in patients with femoral and pelvic fractures, and the risk of DVT is positively correlated with the amount of RBC infusion.

## Introduction

Bone trauma patients are the high-risk population for the formation of DVT, because most of them simultaneously have blood hypercoagulation [1], vascular endothelium injury caused by trauma and surgery, long-term immobilization, and blood stasis caused by edema of surrounding tissues. In the past, the incidence of DVT in patients with lower limb bone trauma without thromboprophylaxis has been as high as 40-60% [2–4]. According to research reports in recent years, the prevention and treatment of DVT have reduced the incidence of above mentioned to 10-20% [5–7]. It is true that more active prevention and treatment have significantly reduced the incidence of DVT compared to the past. However, in the face of this relatively high incidence of DVT, we need to further explore the optimized risk factors for DVT. These factors can be optimized through early identification and timely medical intervention. Perioperative RBC infusion is a factor that needs to be further studied. Currently, there has been controversy regarding the relationship between RBC infusion and perioperative DVT. Most studies have reported that perioperative RBC infusion increases the risk of DVT [8–13], while some researchers have found no correlation between the two events [14–16].

In the previous studies on the correlation between perioperative RBC infusion and DVT, most researchers did not consider the time-event relationship between the two. So, the possibility of a causal relationship between the two events cannot be further investigated. In this study, PSM was used to control confounders related to DVT, and the formation or progression of DVT after RBC infusion was taken as the end point to further analyze the possible causal relationship between RBC infusion and DVT during the perioperative period. Studies on RBC infusion and DVT in patients with current bone trauma also mostly focus on surgical types such as total joint replacement patients [17, 18]. In this study, patients with femoral and pelvic fractures were included, and more attention was paid to the correlation between RBC infusion and DVT in different bone trauma types.

## Materials and methods

### Research objects

Retrospective analysis was performed on patients undergoing surgery for femoral and pelvic fractures in our hospital from May 2018 to December 2019. Inclusion criteria: (1) traumatic fracture; (2) age > 18 years old; (3) complete clinical data. Exclusion criteria: (1) complicated blood system diseases or coagulation dysfunction; (2) long-term history of taking anticoagulant drugs; (3) pregnancy; (4) patients with severe diseases of liver, kidney, heart, brain, and other important organs cannot tolerate surgery; (5) old thrombus history. This study has been approved by the Ethics Committee of the First Affiliated Hospital of Chongqing Medical University (2019-277) and the World Health Organization International Clinical Trial Registry (ChiCTR2000035103).

### Research methods

Clinical data of the patients were collected, and the patients were divided into non-DVT group and DVT group according to the results of preoperative and postoperative deep vein Doppler ultrasound examination of both lower limbs. The DVT group was defined as the development of new postoperative or preoperative thrombus after surgery. Retrospectively analyzed the association between perioperative RBC infusion (from 72 h preoperatively to 72 h postoperatively) and DVT in patients with femoral and pelvic fractures. Perioperative RBC infusion is further divided into four categories: (1) no transfusion; (2) transfusion only before surgery; (3) transfusion only during or after surgery; (4) preoperative/intraoperative/postoperative blood transfusion. After controlling for confounders by PSM, if perioperative RBC infusion was associated with the occurrence of DVT, the formation or progression of DVT after RBC infusion was taken as the end point of the event to further explore the causal relationship between the two.

### Data collection

Clinical data of patients were queried through electronic medical record system and surgical anesthesia system. Preoperative and postoperative pulsed Doppler ultrasonography was performed on both lower limbs using C5-1 linear probe and IU22 system (Philips ATL, Bothwell, WA, USA). Positive criteria for DVT include venous incompressibility, intravascular filling defect, and Doppler signal loss. The formation or progression of perioperative DVT was the endpoint event. The main variables in this study were the time and amount of RBC infusion. The covariates that may be associated with DVT are as follows: gender, age, body mass index (BMI), smoking history, drinking history, and other basic information. Diabetes mellitus, hypertension, coronary heart disease, hyperlipidemia, liver disease, kidney disease, lung disease, malignant tumor, hypoproteinemia, anemia, and other complications. ASA grade, preoperative waiting time (time from trauma to surgery), operative time, tranexamic acid, and anticoagulant therapy.

### Statistical methods

SPSS 26.0 software was used for statistical analysis. Chi-square test or Fisher’s exact probability method was used for counting data, and the results were expressed as percentage (%) to analyze the covariates associated with DVT.

To control the influence of confounding factors, PSM was used to build a logistic model, with perioperative RBC infusion as the dependent variable and DVT-related variable as the covariable. 0.01 was used as caliper value to match the transfusion group and the non-transfusion group in a ratio of 1:1. The matching effect of PSM was evaluated by the standardized difference method, and the standardized difference value (d) was calculated. If d < 0.1, the matching effect was judged to be good.

After matching, the two groups of data were analyzed by binary logistic regression, and the adjusted odds ratio (OR value) and 95% confidence interval (95%CI) were calculated to evaluate the correlation between perioperative RBC infusion and DVT. *P* < 0.05 was considered statistically significant.

## Results

From May 2018 to December 2019, a total of 905 patients underwent lower extremity traumatic fracture surgery in our hospital, of whom 67.51% (611/905) were patients with femoral and pelvic fractures. According to the inclusion and exclusion criteria, 569 patients (93.13%) were included in the study. There were 417 males and 152 females. The mean age was 69.6 ± 19.1 years (20-112 years). With 0.01 as caliper value and 20 factors such as gender, BMI, smoking history, drinking history, diabetes mellitus, hypertension, coronary heart disease, hyperlipidemia, liver disease, kidney disease, lung disease, malignant tumor, hypoproteinemia, anemia, ASA grade as covariates, 126 cases were matched in the transfusion group, and the non-transfusion group at a ratio of 1:1.

### Comparison of DVT correlative covariates between two groups before and after PSM

Before PSM, there were 360 cases (63.27%) in the non-transfusion group and 209 cases (36.73%) in the transfusion group. There were no significant differences in gender, BMI, smoking history, drinking history, diabetes mellitus, hypertension, coronary heart disease, hyperlipidemia, liver disease, kidney disease, lung disease, malignant tumor, and anticoagulant therapy between the two groups (*P* > 0.05). However, there were statistically significant differences between the two groups in age (*P* = 0.002), hypoproteinemia (*P* = 0.000), anemia (*P* = 0.000), ASA grade (*P* = 0.010), operation time (*P* = 0.000), time from trauma to operation (*P* = 0.003), tranexamic acid (*P* = 0.024).

After caliper matching of data between groups by PSM method, 126 cases were found in each group. The above 20 covariates related to DVT were not statistically different between the two groups (*P* > 0.05). It indicated that the data of the two groups were balanced and comparable, as shown in Table 1.

**Table 1:** Distribution characteristics of covariates in patients who received RBC transfusion or did not receive RBC transfusion before and after propensity score matching

Covariates	Before matching						After matching					
	Nontransfusion		Transfusion				Nontransfusion		Transfusion			
	(*N*=360)		(*N*=209)		χ^2^	*P* Value	(*N*=126)		(*N*=126)		χ^2^	*P* Value
Gender												
Male	152	42.20%	76	36.40%	1.890	0.169	44	34.92%	42	33.33%	0.071	0.790
Female	208	57.80%	133	63.60%			82	65.08%	84	66.67%		
Age-group(y)												
≤60	87	24.20%	76	36.40%	12.121	0.002	32	25.40%	35	27.78%	3.400	0.183
60-70	62	17.20%	21	10.00%			22	17.46%	12	9.52%		
>70	211	58.60%	112	53.60%			72	57.14%	79	62.70%		
Drinking history												
No	289	80.30%	172	82.30%	0.350	0.554	110	87.30%	105	83.33%	0.792	0.374
Yes	71	19.70%	37	17.70%			16	12.70%	21	16.67%		
Smoking history												
No	273	75.80%	164	78.50%	0.516	0.473	103	81.75%	101	80.16%	0.103	0.748
Yes	87	24.20%	45	21.50%			23	18.25%	25	19.84%		
BMI (kg/m^2^)												
≤18	47	13.10%	43	20.60%	5.711	0.058	23	18.25%	24	19.05%	1.262	0.532
18-25	245	68.10%	132	63.20%			87	69.05%	80	63.49%		
>25	68	18.90%	34	16.30%			16	12.70%	22	17.46%		
Pulmonary diseases												
No	308	85.60%	167	79.90%	3.062	0.080	101	80.15%	102	80.95%	0.025	0.874
Yes	52	14.40%	42	20.10%			25	19.84%	24	19.04%		
Hyperlipemia												
No	348	96.70%	206	98.60%	1.856	0.853	122	96.83%	124	98.41%	0.171	0.679
Yes	12	3.30%	3	1.40%			4	3.17%	2	1.59%		
Hypertension												
No	254	70.60%	155	74.20%	0.851	0.356	95	75.40%	90	71.43%	0.508	0.476
Yes	106	29.40%	54	25.80%			31	24.60%	36	28.57%		
Coronary heart disease												
No	320	88.90%	189	90.40%	0.333	0.564	114	90.48%	109	86.51%	0.974	0.324
Yes	40	11.10%	20	9.60%			12	9.52%	17	13.49%		
Diabetes												
No	296	82.20%	172	82.30%	0.001	0.982	106	84.13%	104	82.54%	0.114	0.735
Yes	64	17.80%	37	17.70%			20	15.87%	22	17.46%		
Hepatic diseases												
No	292	81.10%	182	87.10%	3.389	0.066	111	88.10%	103	81.75%	1.983	0.159
Yes	68	18.90%	27	12.90%			15	11.90%	23	18.25%		
Renal diseases												
No	307	85.30%	178	85.60%	0.009	0.923	107	84.92%	104	82.54%	0.262	0.609
Yes	53	14.70%	30	14.40%			19	15.08%	22	17.46%		
Malignant tumor												
No	324	90.00%	191	91.40%	0.296	0.586	111	88.10%	112	88.89%	0.039	0.844
Yes	36	10.00%	18	8.60%			15	11.90%	14	11.11%		
Hypoproteinemia												
No	348	96.67%	181	86.60%	22.056	0.000	117	92.86%	117	92.86%	0.000	1.000
Yes	11	3.06%	28	13.40%			9	7.14%	9	7.14%		
Anemia												
No	244	67.80%	63	30.10%	75.385	0.000	55	43.65%	56	44.44%	0.016	0.899
Yes	116	32.20%	146	69.90%			71	56.35%	70	55.56%		
ASA classification												
≤2	94	26.10%	35	16.70%	6.615	0.010	26	20.63%	27	21.43%	0.024	0.877
>2	266	73.90%	174	83.30%			100	79.37%	99	78.57%		
Operative time(h)												
≤2	275	76.40%	83	39.70%	76.236	0.000	72	57.14%	70	55.56%	0.065	0.799
>2	85	23.60%	126	60.30%			54	42.86%	56	44.45%		
Time from trauma to surgery(d)												
<4	193	53.60%	85	40.70%	8.863	0.003	61	48.41%	65	51.59%	0.254	0.614
>4	167	46.40%	124	59.30%			65	51.59%	61	48.41%		
Tranexamic acid												
No	100	27.80%	77	36.80%	5.070	0.024	41	32.54%	44	34.92%	0.160	0.689
Yes	260	72.20%	132	63.20%			85	67.46%	82	65.08%		
Anticoagulation												
No	10	2.80%	9	4.30%	0.957	0.328	3	2.38%	3	2.38%	0.000	1.000
Yes	350	97.20%	200	95.70%			123	97.62%	123	97.62%		

### Equilibrium of the two covariables before and after matching

**Fig. 1:**
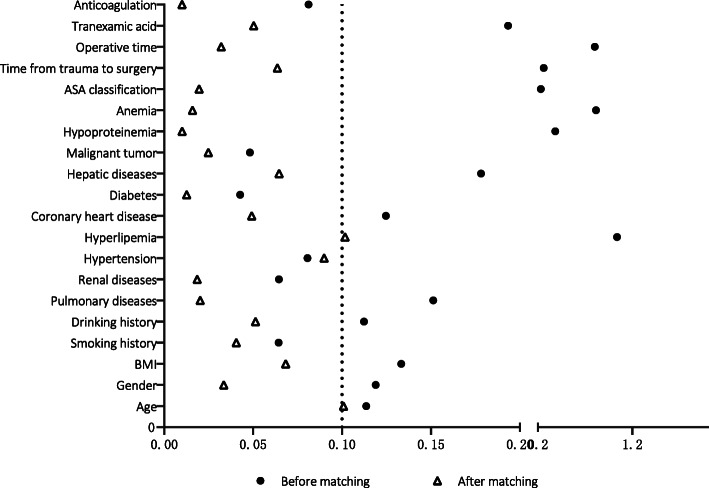
Variable standardization difference diagram

Standardization difference of each covariable between the two groups before and after matching was calculated (d). Before matching, the standardized values of 14 covariates, such as age, sex, pulmonary disease, and hypoproteinemia, were observed between the two groups (d > 0.1). After matching, the d values of 19 covariates were all < 0.1, except for hyperlipidemia (d =0.102). This indicates that PSM has a good matching effect, as shown in Fig. 1.

### Perioperative RBC infusion rate of patients before and after PSM

**Fig. 2:**
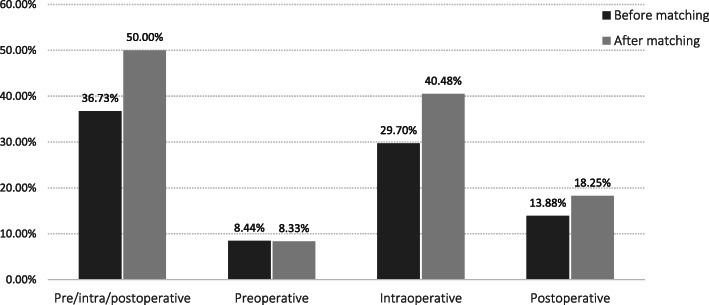
Perioperative red blood cell (RBC) transfusion rate before and after propensity score matching

Among the 569 patients, 36.73% (209/569) received perioperative RBC infusion (from preoperative 72 h to postoperative 72 h), and the rates of preoperative, intraoperative, and postoperative RBC infusion were 8.44% (48/569), 29.70% (169/569), and 13.88% (79/569), respectively. After matching, preoperative, intraoperative, and postoperative RBC infusion rates of 252 patients were 8.33%, 40.48%, and 18.25%, respectively, as shown in Fig. 2.

### Correlation analysis of perioperative RBC infusion and DVT formation before and after PSM

**Fig. 3:**
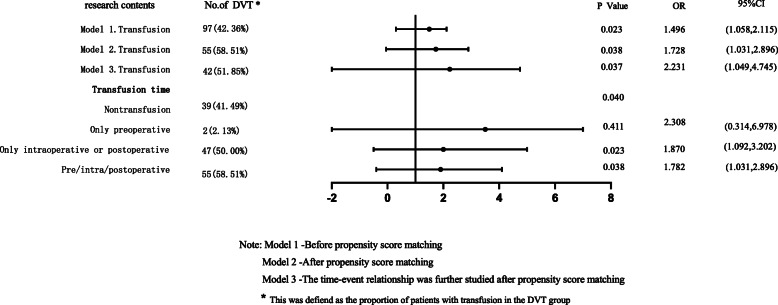
Binary Logistic regression analysis of perioperative RBC transfusion and DVT in patients with femoral and pelvic fractures

Before PSM (model 1), perioperative RBC infusion was associated with the formation of DVT for femoral and pelvic fractures in 569 patients (*P* = 0.023). After PSM (model 2), perioperative RBC infusion was an independent risk factor for the development of DVT in femoral and pelvic fractures (*P* = 0.038), and the risk of DVT in patients with PSM was 1.728 times higher than in patients without RBC infusion. In terms of the timing of transfusion, only intraoperative or postoperative RBC infusion was associated with DVT formation, as shown in Fig. 3.

Further analysis of the total intraoperative or postoperative RBC infusion volume of the patients showed that the perioperative risk of DVT was positively correlated with the transfusion volume. The risk of DVT in patients with RBC infusion of 2-4U and > 4U were 1.833 and 2.667 times higher than ≤ 2U, respectively, as shown in Table 2.

**Table 2:** Correlation analysis of red blood cell infusion volume (intraoperative or postoperative) and DVT after matching

Infusion volume(U)	B	S.E.	Wald	P	OR(95%CI)
≤2			6.887	0.032	1 (Reference)
2-4	0.606	0.443	1.868	0.172	1.833 (0.769,4.372)
≥4	0.981	0.41	5.719	0.017	2.667 (1.194,5.958)

### Analysis of the time-event relationship between perioperative RBC infusion and DVT

**Fig. 4:**
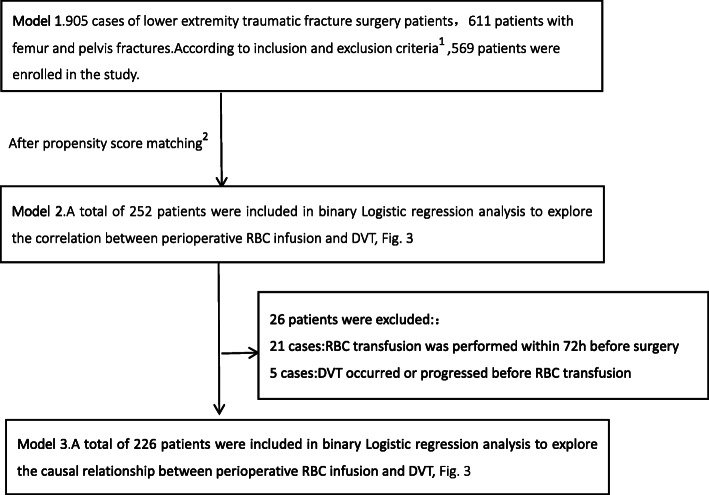
The research route. Note: ^1^ Inclusion criteria: 1. Traumatic fracture; 2. Age > 18 years; 3. Complete clinical data. Exclusion criteria: 1. Combined with hematological diseases or coagulation dysfunction; 2. Long-term use of anticoagulant drugs; 3. Pregnancy; 4. Combined with severe liver, kidney, heart, brain and other important organ diseases cannot tolerate surgery; 5. History of old thrombosis. ^2^ Perioperative RBC infusion was taken as the dependent variable, DVT-related variable as the covariable, and 0.01 as the caliper value. Matching was conducted in a ratio of 1:1

In conclusion, Perioperative RBC infusion is one of the causes of DVT in patients with femoral and pelvic fractures, and the risk of DVT is positively correlated with the amount of perioperative RBC infusion. Among the 252 patients after PSM, we further excluded the patients who received RBC infusion within 72 h before surgery (21 cases), as well as the patients whose DVT occurred or progressed before RBC infusion (5 cases). A total of 226 patients were included, as shown in Fig. 4. The study found that there was still a difference in perioperative RBC infusion between the DVT group and the non-DVT group (*P* = 0.037, OR = 2.231 [95% CI, 1.049-4.745]), as shown in Fig. 3.

## Discussion

Bone trauma patients are at high risk of DVT. A large number of researches have reported the risk factors related to DVT in patients with perioperative bone trauma, such as lower extremity and pelvic fracture, high energy trauma, Glasgow score, prolonged preoperative waiting time, long operative time, advanced age, obesity, and history of malignant tumor [19–21]. The relationship between perioperative RBC infusion and the risk of DVT is controversial. Jackson, Hart, Frisch et al. reported that perioperative RBC infusion does not significantly increase the incidence of venous thromboembolism (VTE) after total joint replacement [14–16]. Rothstein, Helm, Jiang, Goel, Dillon et al. proved that perioperative RBC infusion would increase the risk of VTE in patients undergoing surgery [8–13], such as pediatric surgery [8], ventral hernia repair [9], total knee and hip arthroplasty [10]. It is necessary to conduct PSM analysis of perioperative RBC infusion and DVT. Before PSM, we found that perioperative RBC infusion was associated with the formation of DVT in patients with femoral and pelvic fractures (*P* = 0.023, OR = 1.496 [95% CI, 1.058-2.115]). We further performed PSM for recognized and probable risk factors associated with DVT, as well as sensitivity analysis for confounding variables. We found that this correlation is still valid (*P* = 0.038, OR = 1.728 [95% CI, 1.031-2.896]). Moreover, the risk of DVT in patients with femoral and pelvic fractures who received RBC infusion during perioperative period was 1.728 times higher than that in patients who did not receive RBC infusion. Patients receiving RBC infusion may have one or more DVT risk factors, which may be the reason for the association found in pre-matching data analysis. However, we still reached the same conclusion after controlling the confounders of DVT with PSM, which further demonstrated the independence of correlation between the two.

Although we and some researchers have confirmed that perioperative RBC infusion can increase the risk of DVT in patients undergoing surgery [8–13]. Previous studies did not consider the time-event relationship between transfusion time and DVT events for the two events of RBC infusion and perioperative DVT occurrence or progression. Because of the omission of this detail, even the conclusion that perioperative RBC infusion is related to DVT does not prove a causal relationship between the two. For example, some patients who received RBC infusion before surgery developed postoperative DVT, even if there is no RBC infusion before surgery, it may not be possible to avoid the occurrence of postoperative DVT. Some patients may develop DVT prior to RBC infusion. In order to actually consider the time-event relationship between RBC infusion and the occurrence of DVT during the perioperative period, we take the formation or the progression of DVT as the end point of the event. We used PSM to control confounders and excluded a total of 26 patients from the above two groups. It was found that there was still a difference in perioperative RBC infusion between the DVT group and the non-DVT group (*P* = 0.037, OR = 2.231 [95% CI, 1.049-4.745]). This proves that perioperative RBC infusion is one of the causes of DVT, which is considered to be related to the following aspects: RBC infusion can increase blood viscosity and change local hemorheology, causing erythrocyte aggregation [22]. The increase in the number of red blood cells will lead to platelet aggregation [23]. Storing human RBC under standard blood bank conditions leads to the accumulation of cell-free and microparticle-encapsulated hemoglobin [24], which reacts and removes nitric oxide. This can eventually lead to endothelial injury and vasoconstriction [25, 26]. In addition, most patients with bone trauma are in a state of hypercoagulation and hyperinflammation [1], and blood transfusion can induce inflammation, which may further lead to the formation of postoperative thrombus in patients [27]. In terms of transfusion timing, we divided perioperative RBC infusion into four categories: (1) no transfusion; (2) transfusion only before surgery; (3) only intraoperative or postoperative blood transfusion; (4) pre/intra/postoperative blood transfusion. This study found that only intraoperative or postoperative RBC infusion was associated with the occurrence of DVT (*P* = 0.023, OR = 1.870 [95% CI, 1.092-3.202]), and only preoperative RBC infusion was not associated with DVT (*P* = 0.411). However, the number of preoperative RBC infusion cases was small, and whether preoperative RBC infusion is related to DVT remains to be further studied. We also statistically analyzed the intraoperative or postoperative RBC infusion volume of patients, and found that the risk of DVT in patients with RBC infusion 2-4U and > 4U were 1.833 and 2.667 times higher than ≤ 2U, respectively. The previous retrospective cohort study of 1233 trauma patients by Meizoso et al. using RAP score found that RBC infusion of more than 4 units was an independent risk factor for DVT [19]. Although we classified the amount of transfused red blood cells differently with Meizoso’s study, our conclusions were consistent with those of Meizoso, Goel, and Dillon et al. [12, 13, 19]. The risk of perioperative DVT was positively correlated with the transfusion volume, which is also one of the reasons for us to more strictly control the indications of blood transfusion.

Perioperative RBC infusion strategy depends on the comprehensive evaluation of patients’ general conditions, traumatic conditions, surgery, and other factors. In our hospital, transfusion of RBC is usually performed for patients with preoperative anemia, intraoperative and postoperative massive blood loss. The systematic review and meta-analysis of Gu showed that the perioperative erythrocyte infusion rate in orthopedic trauma patients was 1-12% [28]. Taking hip fracture patients as an example, it has been reported in recent years that the perioperative RBC infusion rate is 11-45% [29]. The preoperative, intraoperative, and postoperative blood transfusion rates of patients with femur and pelvis fractures in our hospital were 8.44%, 29.70%, and 13.88%, respectively. The relatively high rate of intraoperative blood transfusion may be related to the fact that most of the patients in our hospital are elderly, with many complications, and most of them are transported by subordinate hospitals without surgical control of post-traumatic bleeding. Cao, Song, Slover et al. reported that risk factors for perioperative blood transfusion in patients with bone trauma included female, older age, smaller BMI, ASA classification ≥ 3, preoperative anemia, prolonged operation time, more intraoperative bleeding caused by no tranexamic acid, no tourniquet, type of operation, and more complications including myocardial infarction, congestive heart failure, peripheral vascular disease, cerebrovascular disease, and chronic obstructive pulmonary disease [17, 30, 31]. In this study, we regarded gender, age, BMI, smoking history, drinking history, diabetes, hypertension, coronary heart disease, hyperlipidemia, liver disease, kidney disease, lung disease, malignant tumor, hypoalbuminemia, anemia, ASA classification, preoperative waiting time, trauma to the operation time, operation time, use of tranexamic acid, and other 20 factors as confounding factors and we controlled for bias for these factors. Almost all of the above risk factors for perioperative RBC infusion were included in the confounding factors of this study, which also increases the reliability of the results of this study.

Patients with severe bone trauma may face the risk of hemorrhagic shock caused by fracture, acute intraoperative massive hemorrhage, and postoperative hematoma formation, etc. These patients may not be able to avoid transfusion of RBC during the perioperative period. RBC infusion may also lead to adverse consequences such as prolonged hospital stay, increased medical costs, cardiopulmonary complications, sepsis, multiple organ failure, and even death [32, 33]. Current guidelines emphasize preoperative evaluation of trauma patients while weighing the risks and benefits of blood transfusion, and the use of adjuvant agents such as antifibrinolytic drugs to limit perioperative bleeding and secondary complications [34, 35]. Due to the simultaneous risk of bleeding and thrombosis, the American guidelines for perioperative blood transfusion and adjuvant therapy in anesthesiology refer to patients’ preoperative preparation including discontinuation or alteration of anticoagulant therapy [34]. The ACCP guidelines state that anticoagulant therapy should be carefully considered to avoid increasing the risk of bleeding in the following situations including any fatal bleeding, bleeding into a critical organ (e.g., retroperitoneal, intracranial, intraocular, or intraspinal), clinically overt (e.g., GI) bleeding associated with a ≥ 2 g/dL drop in hemoglobin level or requiring ≥ 2 units of blood transfused, and bleeding leading to reoperation [4]. According to the actual situation of our hospital, anticoagulant therapy will be delayed or interrupted, or even not started, for patients with bone trauma combined with the above conditions. In addition to formulating individualized anticoagulant prevention and treatment plans, optimizing the perioperative blood transfusion strategy may also be a measure to reduce the risk of DVT. Minimizing the time from admission to surgery, using non-transfusion substitutes such as iron to improve preoperative hemoglobin levels, optimizing surgical methods and improving surgical skills to limit intraoperative bleeding may reduce unnecessary RBC infusion. Considering the correlation between RBC infusion and DVT, especially for patients with bone trauma combined with DVT risk factors, we may need to more strictly grasp the indications of RBC infusion, and at the same time, we should strengthen the screening and prevention of DVT for such patients receiving RBC infusion.

The formation of DVT is a complex process, which is affected by many unknown factors. Compared with most previous studies on the risk factors of perioperative DVT in patients with bone trauma, this study used PSM and Logistics regression analysis to explore the correlation between perioperative RBC infusion and DVT in patients with femoral and pelvic fractures, reducing the confusion bias in the study. This achieved a balanced distribution of covariates between the case group and the control group to achieve a similar effect to prospective randomized controlled trials [36]. In addition, two types of patients who received preoperative RBC infusion and those whose DVT occurred or progressed before RBC infusion were excluded in this study. Our aim was to explore the time-event relationship between perioperative RBC infusion and DVT, and also to confirm the causal relationship between the two. This has not been reported in previous studies. Although we used PSM to control the bias caused by 20 confounding factors, this study also has some limitations: There may be potential risk factors that were not included in our PSM analysis, and these unknown confounding variables would have an impact on the outcome of the study. This study was a single-center retrospective study, and more multi-center prospective randomized controlled trials are needed in the future to further explore the relationship between perioperative blood transfusion and DVT.

## Conclusion

Perioperative RBC infusion is one of the causes of DVT in patients with femoral and pelvic fractures, and the risk of DVT is positively correlated with the amount of perioperative RBC infusion. DVT screening and individualized prevention and treatment should be emphasized in patients with bone trauma who receive RBC infusion. Further studies are needed to investigate the effective prevention and treatment of perioperative reasonable blood transfusion and DVT in patients with lower limb bone trauma.

## Data Availability

All the data will be available upon motivated request to the corresponding author of the present paper.

## Abbreviations

RBC: Red blood cell
DVT: Deep vein thrombosis
PSM: Propensity score matching
BMI: Body mass index
ASA: American Society of Anesthesiologists
VTE: Venous thromboembolism
